# Meat Consumption and Availability for Its Reduction by Health and Environmental Concerns: A Pilot Study

**DOI:** 10.3390/nu15143080

**Published:** 2023-07-08

**Authors:** Andrea Turnes, Paula Pereira, Helena Cid, Ana Valente

**Affiliations:** 1 ATLANTIC—University Institute, 2730-036 Barcarena, Portugal; 2 Egas Moniz Interdisciplinary Research Center, Egas Moniz School of Health and Science, Quinta da Granja-Campus Universitário, 2829-511 Monte da Caparica, Portugal; 3 HeartGenetics, Genetics & Biotechnology, Av. Prof. Gama Pinto n°. 2, 1649-003 Lisbon, Portugal; 4 Ecogenetics and Human Health Research Group, Environmental Health Institute (ISAMB), Associate Laboratory TERRA, Faculty of Medicine (FMUL), University of Lisbon, 1649-028 Lisbon, Portugal

**Keywords:** meat, consumption, health, environment, ecological footprint

## Abstract

(1) Background: Excessive meat consumption has raised multiple health and environmental concerns; however, there are no data on the population’s willingness to reduce its intake for these reasons. The current study aims to assess the frequency of meat intake and readiness to limit consumption due to concern about the impact on health and the environment in residents of the Lisbon metropolitan region. (2) Methods: This analytical cross-sectional observational study was carried out in 197 residents in the metropolitan region of Lisbon. The participants were divided into two groups by age (GI: 20–29 years; GII: 40–64 years). Meat consumption and willingness to reduce it were assessed through a questionnaire. (3) Results: Most participants (67%) reported not having knowledge about the ecological footprint of meat. Being a less frequent meat consumer (<1 time per day) is associated with a willingness 3.6 times higher (*p* < 0.001) to reduce meat consumption due to sensitivity to the impact on health and 4.0 times higher (*p* < 0.001) due to environmental reasons. (4) Conclusions: Lower meat consumption frequency was associated with reductions in this consumption for environmental and health reasons.

## 1. Introduction

In Portugal, dietary imbalances are evident [1,2,3], where the consumption of meat, sugar, fat, and salt is much higher than recommended [4]. Changes in eating habits over the years have been remarkable [5]. According to recent data, just 26% of the Portuguese population adheres to the Mediterranean diet [6], indicating a noteworthy divergence from this dietary pattern known globally as a health promoter. Some studies have suggested a link between frequent and high meat consumption and noncommunicable diseases (such as cancer, diabetes, and cardiovascular disease) [7,8,9,10]. Indeed, food choices, together with other lifestyle behaviors such as smoking and lack of physical activity, have been identified as risk factors for one-third of all fatalities in Portugal [11].

More recently, environmental concerns have also been raised about excessive meat consumption [1,4,12], especially when referring to red meat, which appears to have the higher ecological footprint [13]. The emission of greenhouse gases is 57 times higher in beef when compared with tofu, while for poultry it is 4 times higher [13]. Even though there are no statistics on public knowledge of these data, this information is freely available to the public via the Our World in Statistics website. Also, the results of Sanchez-Sabaté and colleagues (2019) have shown that meat consumers are not willing to change their dietary habits for environmental reasons [12].

In Portugal, the Food Balance report published by the National Institute of Statistics for the period 2016–2020 showed an increase in meat consumption in this period, despite a decrease during the pandemic, but still 8.7% higher than the previous analysis period of 2012–2015 [1]. Currently, Portuguese society is undergoing a transformation; Europe is experiencing an energy crisis, which will certainly affect food supply, with predicted increases in meat prices and a grain shortage [14]. The pressure to modify eating habits for economic reasons is great, and this could be a chance to promote public knowledge about the need to reduce meat consumption, which has obvious health and environmental benefits. The theme of the current piece is highly timely, given the current economic downturn, social unrest, energy problem, and environmental consciousness [15]. According to current knowledge, this is the first study in Portugal that aimed to assess the frequency of meat consumption and willingness to reduce consumption due to concern about the impact on health and the environment in residents of the Lisbon metropolitan region.

## 2. Materials and Methods

### 2.1. Research Design and Sample

This cross-sectional analytical study was conducted on a sample of Lisbon metropolitan region inhabitants. From a 2,264,004 [16] population of individuals who were at least 19 years old, a minimum sample size of 97 individuals was considered with a 95% confidence level and a 10% margin of error [17]. The study was conducted from November 2022 to March 2023.

Sample Selection, Inclusion, and Exclusion Criteria

The current study used a stratified probabilistic sample with the following inclusion criteria: (1) be between the ages of 20 and 29 or 40 and 64, (2) live in the Greater Lisbon region, and (3) have an email address to obtain the self-completion questionnaire to be used in the study.

Individuals younger than 20 years old and 64 years old or older were eliminated, as were individuals who could not read or write and did not have e-mail access. Only citizens of the Lisbon metropolitan region were considered.

### 2.2. Variables

Several categories of variables were created:

- General and lifestyle variables: (1) age, (2) sex, (3) practice of physical exercise, (4) consumption of alcoholic beverages, (5) tobacco consumption, and (6) presence of chronic diseases.
- Socioeconomic variables: (1) place of residence, (2) household size, (3) literary qualifications, and (4) professional situation.
- Dietary variables: (1) frequency of meat consumption, (2) motivation for meat consumption, (3) willingness to change meat consumption habits due to knowledge of the associated ecological footprint, and (4) willingness to change meat consumption habits due to knowledge of the negative effects on health.

### 2.3. Measuring Instrument and Techniques

With the help of an online form maker [18], a questionnaire with 17 closed-ended questions was created and applied. In the beginning of the questionnaire, the project and its main goal were described. This was followed by a question in which the participant agrees to take part in the study and gives permission for the data to be used for that study. Eleven of the questions on the survey were about general information and how people live. Two questions were about how often and why people eat meat, and three questions were about why people want to change their eating habits because they know about their impact on the environment and want to improve their health. The questionnaire was sent to and shared within six companies in the Lisbon urban area that work in different fields. In these companies, data collection was performed to obtain results that were more diverse and more representative of the people who live in Lisbon.

### 2.4. Statistical Analysis

An operationalization table of the variables under study was constructed, in which the codification, description, valuation, and statistical categorization of the variables were carried out. The referred table was used as a tool for the construction of a database that allowed the compilation of all the results of the variables under study. The statistical analysis was performed using the statistical software for Windows, SPSS^®^, version 26.0 (SPSS Inc., Chicago, IL, USA). The results were expressed as a number and percentage. The frequency distribution of the qualitative variables was evaluated using the chi-square test and, in the presence of statistical significance, the Z test for proportions was applied. Contingency tables were also created, and the Mantel Haenszel test was applied to estimate associations between qualitative variables with differences in frequency distribution. For all tests, statistical significance was considered when *p* < 0.05.

## 3. Results

Table 1 displays the general characteristics of the sample across two distinct age groups. The sex distribution in the field of GI showed that approximately 29% of individuals identified as male, while the remaining 71% identified as female. The GII data revealed a comparatively greater proportion of female individuals within the distribution. The study did not identify any statistically significant variations between the groups with respect to the following variables: sex, household size, existence of chronic illness, existence of a chronic illness that restricts meat consumption, tobacco use, alcohol consumption, and frequency of physical activity of at least twice per week. Overall, the sample population exhibited a majority of over 50% of individuals with a household comprising three or more members. Approximately 84% of the participants reported the absence of any chronic medical conditions. With respect to smoking behaviors, a majority of 75.6% of respondents indicated that they did not engage in smoking. The data revealed that 44.2% of adults reported consuming alcohol at least once per week. Most of the participants, specifically 53.3%, reported being sedentary as they disclosed not engaging in physical exercise for a minimum of two times per week.

The analysis found significant differences (*p* < 0.001) in educational qualifications between the groups. A higher percentage of individuals in GII (62.0%) had higher education compared with GI (38.0%), while a higher percentage of participants in GI (70.7%) had completed secondary education compared with GII (29.3%). The statistical significance of professional status results was observed (*p* < 0.001). The distribution of employees in GI (33.3%) and GII (66.7%) and students in GI (87.5%) and GII (12.5%) showed significant differences.

The characterization of the sample by sex is shown in Table 2. Of the 66 male participants, 27 were aged between 20 and 29 (GI) years and 39 between 40 and 64 years (GII). Regarding females, 67 of the participants belonged to GI and 64 to GII. No statistically significant differences were found for the variables: household size, educational qualifications, professional situation, presence of chronic disease, presence of chronic disease that limits meat consumption, tobacco consumption, and frequency of physical exercise at least two times per week. Significant differences (*p* < 0.001) were found in relation to alcohol consumption at least once a week, and this consumption was verified in more than half of the participating males (42) and only in 45 females.

Table 3 shows the frequency of meat intake and readiness to limit consumption by group. According to the findings, no statistically significant differences were found between the groups in terms of meat consumption frequency, reasons for never consuming meat, or willingness to reduce consumption due to health and environmental concerns. Knowledge of the ecological footprint was similarly comparable across groups. The frequency of meat consumption four to six times per week was most frequently stated by individuals (86), with 38 in GI and 48 in GII. The option of consuming more than six times per week was the second most popular, with 53 people indicating this frequency of intake, 31 in GI and 22 in GII. Only 15 people said they would never eat meat again, and the most common explanation was that they were devout vegetarians. In terms of willingness to minimize meat eating, 139 people said they were willing to do so because they were concerned about the influence on their health. This option was chosen by 66 people in GI and 73 people in GII. A large proportion of participants (67%) stated that they were unaware of the ecological footprint of meat. However, 125 (GI: 57 vs. GII: 68) reported being able to minimize their consumption due to environmental concerns.

The frequency of meat consumption and willingness to reduce this consumption by sex are presented in Table 4. No significant differences were observed between males and females regarding the frequency of meat consumption, reasons for never consuming meat, and knowledge about the ecological footprint. The frequency of meat consumption four to six times per week was again the most described by both sexes, with 34 of the 66 males evaluated consuming meat four to six times per week. Only one male indicated never eating meat, while 14 females chose this frequency. In addition, 57.8% of females indicated that they were unaware of the ecological footprint of meat, and 42.2% of the males indicated the same. Statistically significant differences were observed in terms of willingness to reduce meat consumption due to its impact on health, with females be more willing (73.9%) to make this reduction than males (26.6%). The same was verified in relation to the willingness to reduce meat consumption based on knowledge of the environmental impact (*p* < 0.001), in which 74.4% of females responded that they were available to reduce meat consumption against 25.6% of males.

Table 5 demonstrates the relationship of the availability to lower the frequency of meat eating in participants who consume it more than six times per week. The findings revealed that less frequent consumers, or those who consume meat less than once a day, are substantially (*p* < 0.001) more likely (79.1%) to reduce their consumption owing to health concerns than the most frequent consumers (20.9%).

Being a meat consumer less than once a day relates to a 3.6-fold greater readiness to reduce meat eating due to health sensitivity. When it comes to the willingness to reduce meat consumption due to environmental concerns, less frequent consumers (consumption one time per day) are more willing to do so (81.6%) than the most frequent consumers (daily consumption), who only have 18.4% of affirmative answers. Due to environmental sensitivity, being a less frequent consumer than daily relates to a 4.0 times greater desire to cut meat intake.

Table 6 shows the results of the association by sex of availability to reduce the frequency of meat consumption in the most frequent consumers (more than six times per week). This difference was statistically significant (*p* < 0.001). Being a meat consumer daily and being a female seem to be associated with an eight times greater willingness to reduce meat consumption due to sensitivity to the impact on health than being a male and a frequent meat consumer (one time per day). Regarding availability to reduce meat consumption due to sensitivity to environmental impact, it was found that females more frequently reported being available to reduce meat consumption (69.6%) compared with males (30.4%). Being a meat consumer daily and being a female seem to be associated with a 6.4 times greater availability to reduce meat consumption due to sensitivity to the impact on health than being a male and a frequent meat consumer (one time per day).

## 4. Discussion

According to current knowledge, this is the first study in Portugal to examine the frequency of meat intake and sensitivity to the impact on health and the environment in residents of the Lisbon metropolitan region. Food sustainability and healthy eating are widely disseminated through various media to influence habits and, as a result, lessen the impact on health and the environment with more aware choices [19]. Excessive meat consumption has negative health repercussions as well as a significant ecological imprint, and dietary modifications are becoming increasingly important [9,20].

The findings revealed no differences in sensitivity to the ecological footprint between groups I and II, which were formed based on various age ranges. It was expected to find differences between the studied groups, and our findings were consistent with those presented in a study conducted in several European Union countries [21] in which the goal was to evaluate the possible presence of differences in sensitivity to the ecological footprint and meat consumption in two well-differentiated age ranges (GI: 20–29 years and GII: 40–64 years old).

We anticipated that the younger group would have better environmental awareness and sensitivity to lowering meat intake due to the negative environmental and health implications than GII. GI has different cultural, political, and gastronomic influences than GII because it is made up of younger elements. Because they were older, the persons in GII may have been more exposed to more unpredictability in food availability throughout their lives than the subjects in GI, who were from a younger generation. Participants in GII were most likely subject to food policies in Portugal that were based on assuring food accessibility until they reached maturity. GI individuals, on the other hand, were from an age in which food regulations began to prioritize nutritional status and health promotion [22].

The current study found no significant differences between the two groups evaluated in Portugal regarding their knowledge of the ecological footprint of meat. A high percentage of participants, regardless of age or sex, indicated that they did not have this knowledge. Despite the different influence of food policies in Portugal on the two evaluated groups, the present study found no significant differences concerning this knowledge. This fact was somewhat unsettling because it showed the need for improvements in communication and the perception of information on the part of the customers whose responses were analyzed, as well as maybe additional consumers from the Portuguese population. There were no discernible differences regarding knowledge regarding the ecological footprint of meat among age groups or between the sexes, and there are no comparative studies on this topic that have been conducted in Portugal. It is also important to note that the evaluated sample had a large representation of participants with secondary or higher education. Although it was anticipated that participants with more educational qualifications would have a greater knowledge about the ecological footprint of meat, the results did not show any differences between the groups based on this variable [6,23,24].

It was also observed that those who consumed meat one to three times per week were more frequently in GII, whereas participants who consumed meat more than six times per week daily were more frequently in GI. The fact that the differences identified were not statistically significant demonstrates that the age range does not appear to have any bearing on the number of times that people consume meat. These results were not what was expected because there has been a growing trend among young people to become aware of the ecological footprint of meat. This information has been widely disseminated on the internet [25] and in other media, as well as the promotion of the Mediterranean dietary pattern [26], which is based on promoting the consumption of vegetable protein to the detriment of animal protein. Arnaudova et al. (2022) [27], who analyzed the frequency of meat intake in university students, did not find any significant differences by age or sex in their findings. The findings of the current study agreed with the findings of the research work by Arnaudova et al. (2022) [27]. The research conducted by Verain and Dagevos (2022) [24], which described and compared people who consume meat, lends support to the notion that the primary motive of people who do not consume meat is concern for the welfare of animals, with health and sustainability coming in a distant second and third place, respectively. Another study was carried out in countries of the European Union [21], which investigated whether reducing meat consumption would be associated with the concept of “eating a healthy and sustainable diet.” The authors concluded that in southern Europe, the respondents do not associate reducing meat consumption as an active positive behavior toward healthy eating and/or a contribution to the sustainability of the planet.

In the current analysis, there were not found any statistically significant variations between age groups regarding the desire to limit the amount of meat consumed. On the other hand, when looking at the complete sample, it was discovered that a greater number of participants (*n* = 139) indicated that they were willing to cut their consumption of meat due to concerns regarding its influence on health as opposed to concerns regarding its impact on the environment (*n* = 63). When the examination of availability to reduce meat consumption was carried out by sex, disparities were discovered. It was found that females were more likely to be available to reduce this consumption due to their sensitivity to the influence that it had on both their health and the environment. On the other hand, it was discovered that this availability was greater for reasons related to health (OR = 8.0; *p* < 0.001) than it was for reasons related to the environment (OR = 6.4; *p* < 0.001). A study that was conducted out on 713 German adults with diverse patterns of meat consumption, published by Verain and Dagevos (2022) [24], reveals that males have more resistance to reducing their meat intake, possibly due to a lack of awareness, a lack of interest, or cultural reasons. According to the findings of other studies [23,28], females are more aware of the effects of human activity on the environment, whereas males are less worried about environmental issues. Carvalho and Li (2009) [29] conducted a research study on the lack of desire by males to reduce their intake of meat. The authors concluded that this lack of motivation may be related to males having a weaker view of their own health status in comparison with females.

In the current study, it was shown that individuals who ate meat less frequently (less than once per day) reported being more open to reducing their consumption of meat when compared with individuals who ate meat more frequently. These results were observed for sensitivity to both the impact on health and the environment. However, less frequent consumers were 3.6 times more likely (*p* < 0.001) to reduce their consumption of meat due to their awareness of the negative impact it had on their health and 4.0 times more likely (*p* < 0.001) to do so for environmental reasons. There are several studies that can be found in the scientific literature that discuss a variety of arguments in favor of lowering one’s consumption of meat. Others refer to sensitivity to health impacts as the primary cause [24,30], and the price of meat has also been seen as one of the key reasons for reducing its consumption [30]. Some of the reasons have to do with animal welfare [12,31], while others point to sensitivity to health impacts as the main reason [24,30].

In a study that was conducted [32] in Canada, the impact of community interventions on changing eating habits was analyzed, and the conclusion reached was that an individual’s level of knowledge alters the tendency to internalize the message and subsequent action in reducing meat intake. The study was carried out to evaluate the impact of community interventions on changing eating habits. This research also focused on the significance of making alternative food selections after reducing the amount of meat consumed, and it implied that these shifts did not necessarily have positive effects on the environment. It is of the utmost importance that community actions be suited to the target audience that is intended to be affected, and to do this, it is vital to know the population and differentiate between the groups within it. It is also important to educate people so that they may make informed decisions about the dietary alternatives that will help them reduce their meat consumption while also benefiting the environment. Because eating meat has such a significant negative effect on both human health and the natural world, adopting new eating patterns and making more conscientious and environmentally friendly decisions is more important than ever. One of the first stages toward accomplishing this objective is being familiar enough with the people to be able to intervene in a forceful manner [1,9,12,20,31,32].

It will also be important to consider the possible effects of seasonality on meat consumption in Portugal. Although the study was conducted from November 2022 to March 2023, the questionnaire was applied from the end of January until March. Thus, the influence of the holiday season was not confirmed. In Portugal, the availability of some foods may be influenced by seasonality, as with fruit, vegetables, and fish, but the tables of seasonality and food availability in the national market do not include meat [33]. It is also important to point out that based on the latest results of the Food Balance Sheet for the five-year period (2016–2020), its availability has increased by 8.7% (+6.7 kg/inhabitant) in relation to the homologous previous period, currently being the group of the food wheel with the greatest deviation of availability for consumption in relation to the recommended daily consumption [1]. Thus, since 2012, meat consumption in Portugal has been three times higher (16.4%) than recommended (5%) on the food wheel and with little seasonal variation.

The price of meat is also a possible factor contributing to its lower daily consumption. Food price inflation between November 2022 and March 2023 was quite high [34]. The foods that had the most significant increases between February and March 2023 were (1) dairy products (+27.3%), (2) fish (+27%), (3) grocery products (+25%), (4) fruits and vegetables (+24.7%), and meat (+22.8%) [35]. The increase in the price of meat and other foods described is a factor that may have contributed to a lower consumption of meat due to a decrease in the consumer’s purchasing power. However, the results of the III Great National Sustainability Survey [36], presented in October 2022, had already shown a significant reduction in meat consumption (−32%) compared with 2018 (pre-pandemic). Thus, the effect of the pandemic seems to have had a greater impact on reducing meat consumption than the price increase since several foods that can be protein alternatives to meat had an even greater increase (e.g., fish, dairy products, and grocery stores). Furthermore, during periods of greater financial trouble, consumers also change their meat buying patterns, favoring types of meat with lower prices per kilogram (e.g., pork meat).

### Study Limitations

The current study had a few flaws and restrictions. The validated questionnaires on this topic referred to consumption frequencies and/or eating habits; however, no validated questionnaire on the subject being studied was found in the scientific literature. Consequently, it was necessary to create a new questionnaire. Although the sample size was representative of the population in the sense that it was larger than the minimum sample size associated with a confidence level of 95% and a margin of error of 10%, the authors intend to continue this work in the future and increase the number of participants to decrease the error to 5%, which would require 385 participants in each group. Although the questionnaire was administered without any kind of personal identity record, there was still a possibility that there was a bias related to social desirability and participation. This was another drawback of the study. Very few of the participants in this study had even completed their primary or secondary education, which meant that the work did not adequately reflect people with lower levels of literary qualifications. It will be necessary in the future to carry out an evaluation that also includes a greater number of participants with characteristics that are inadequately reflected in the work that has already been performed on the research. Another aspect that was not considered was related to the amount of meat consumed since only the frequency of consumption was evaluated. It will be necessary to incorporate a methodology for assessing the food portion of meat consumed in combination with the frequency of consumption into the work that will be performed in the future.

## 5. Conclusions

This study contributed to the evaluation of the frequency of meat consumption, the knowledge of the ecological footprint, and the availability of participants to reduce their meat consumption because of sensitivity to the impact on health and the environment, among residents of the metropolitan region of Lisbon. Regardless of age or sex, most participants said that they lacked understanding regarding the ecological footprint of meat consumption. There was no discernible difference in the frequency of meat eating based on age or sex. Less frequent meat consumers (consumption of less than once per day) were more available to minimize their meat consumption than more frequent meat consumers (consumption of more than six times per week) because they were more sensitive to the influence that their behavior may have on their health or the environment. When it came to people who ate meat more than six times a week, females were more available than males to cut their consumption of meat if doing so would have a positive effect on their health or the environment.

Additional research is required to properly analyze the prevalence of meat intake and the options available to lessen that prevalence. In the future, it is planned to continue the inquiry into this topic, with the goals of increasing the sample size, expanding the analyzed geographic area, including additional potential variables that may motivate people to consume less meat, and quantifying the food portion that was consumed.

## Figures and Tables

**Table 1:** Characterization of the sample by group.

Characteristics	GI (*n* = 94)	GII (*n* = 103)	Total (*n* = 197)	*p*
Sex	94 (47.7)	103 (52.3)	197 (100)	
Masculine	27 (40.9)	39 (59.1)	66 (100)	0.175
Feminine	67 (51.1)	64 (48.9)	131(100)	
Household size	94 (47.7)	103 (52.3)	197 (100)	0.068
1 person	14 ^a^ (51.9)	13 ^a^ (48.1)	27 (13.7)	
2 persons	23 ^a^ (63.9)	13 ^b^ (36.1)	36 (18.3)	
3 persons	29 ^a^ (51.8)	27 ^a^ (48.2)	56 (28.4)	
4 persons	18 ^a^ (35.3)	32 ^b^ (64.7)	51 (25.9)	
>4 persons	10 ^a^ (37.0)	17 ^a^ (63.0)	27 (13.7)	
Literary abilities	94 (47.7)	103 (52.3)	197 (100)	<0.001 *
2nd and 3rd cycle of basic education	1 ^a^ (50.0)	1 ^a^ (50.0)	2 (1.0)	
High school	41 ^a^ (70.7)	17 ^b^ (29.3)	58 (29.4)	
University education	52 ^a^ (38.0)	85 ^b^ (62.0)	137 (69.5)	
Professional situation	94 (48.2)	101 (51.8)	195 (100)	<0.001 *
Employee	37 ^a^ (33.3)	74 ^b^ (66.7)	111 (56.9)	
Student	28 ^a^ (87.5)	4 ^b^ (12.5)	32 (16.4)	
worker—student	27 ^a^ (56.3)	21 ^a^(43.8)	48 (24.6)	
unemployed	2 ^a^ (50.0)	2 ^a^ (50.0)	4 (2.1)	
Presence of chronic illness	93 (47.4)	103 (52.6)	196 (100)	0.105
Yes	11 (34.4)	21 (65.6)	32 (16.3)	
No	82 (50.0)	82 (50.0)	164 (83.7)	
Presence of chronic illness that limits meat consumption	11 (34.4)	21 (65.6)	32 (100)	0.968
Yes	1 (33.3)	2 (66.7)	3 (9.4)	
No	10 (34.5)	19 (65.5)	29 (90.6)	
Smoke at least one time per week	94 (47.7)	103 (52.3)	197 (100)	0.974
Yes	23 (47.9)	25 (52.1)	48 (24.4)	
No	71 (47.7)	78 (52.3)	149 (75.6)	
Consume alcoholic beverages at least once a week	94 (47.7)	103 (52.3)	197 (100)	0.313
Yes	38 (43.7)	49 (56.3)	87 (44.2)	
No	56 (50.9)	54 (49.1)	110 (55.8)	
Exercise at least twice a week	94 (47.7)	103 (52.3)	197 (100)	0.753
Yes	45 (48.9)	47 (51.1)	92 (46.7)	
No	49 (46.7)	56 (53.3)	105 (53.3)	

The results are expressed as the number of individuals (percentage). * Statistically significant (*p* < 0.05). Frequencies in the same line marked with different letters (a,b) are statistically different according to the Z test for proportions (*p* < 0.05).

**Table 2:** Characterization of the sample by sex.

Characteristics	Male (*n* = 66)	Female (*n* = 131)	Total (*n* = 197)	*p*
Groups	66 (33.5)	131 (66.5)	197 (100)	0.175
GI: 20–29 (years)	27 (28.7)	67 (71.3)	94 (47.7)	
GII: 40–64 (years)	39 (37.9)	64 (62.1)	103 (52.3)	
Household size	66 (33.5)	131 (63.5)	197 (100)	0.462
1 person	13 (48.1)	14 (51.9)	27 (13.7)	
2 persons	11 (30.6)	25 (69.4)	36 (18.3)	
3 persons	16 (28.6)	40 (71.4)	56 (28.4)	
4 persons	16 (31.4)	35 (68.6)	51 (25.9)	
>4 persons	10 (37.0)	17 (63.0)	27 (13.7)	
Literary abilities	66 (33.5)	131 (66.5)	197 (100)	0.759
2nd and 3rd cycle of basic education	1 (50.0)	1 (50.0)	2 (1.0)	
High school	21 (36.2)	37 (63.8)	58 (29.4)	
University education	44 (32.1)	93 (67.9)	137 (69.5)	
Professional situation	66 (33.8)	129 (66.2)	195 (100)	0.569
Employee	41 (36.9)	70 (63.1)	111 (56.9)	
Student	10 (31.3)	22 (68.8)	32 (16.4)	
Worker—student	13 (27.1)	35 (72.9)	48 (24.6)	
Unemployed	2 (50.0)	2 (50.0)	4 (2.1)	
Presence of chronic illness	66 (33.7)	130 (66.3)	196 (100)	0.617
Yes	12 (37.5)	20 (62.5)	32 (16.3)	
No	54 (32.9)	110 (67.1)	164 (83.7)	
Presence of chronic illness that limits meat consumption	12 (37.5)	20 (62.5)	32 (100)	0.273
Yes	2 (66.7)	1 (33.3)	3 (9.4)	
No	10 (34.5)	19 (65.5)	29 (90.5)	
Smoke at least one time per week	66 (33.5)	131 (66.5)	197 (100)	0.305
Yes	19 (39.6)	29 (60.4)	48 (24.4)	
No	47 (31.5)	102 (68.5)	149 (75.6)	
Consume alcoholic beverages at least once a week	66 (33.5)	131 (66.5)	197 (100)	<0.001 *
Yes	42 (48.3)	45 (51.7)	87 (44.2)	
No	24 (21.8)	86 (78.2)	110 (55.8)	
Exercise at least twice a week	66 (33.5)	131 (66.5)	197 (100)	0.957
Yes	31 (33.7)	61 (66.3)	92 (46.7)	
No	35 (33.3)	70 (66.7)	105 (53.3)	

The results are expressed as the number of individuals (percentage). * Statistically significant (*p* < 0.05).

**Table 3:** Assessment of meat consumption frequency and willingness to reduce this consumption by group.

Characteristics	GI (*n* = 94)	GII (*n* = 103)	Total (*n* = 197)	*p*
Frequency of meat consumption	94 (47.7)	103 (52.3)	197 (100)	0.237
Never	8 (53.3)	7 (46.7)	15 (7.6)	
One to three times per week	17 (39.5)	26 (60.5)	43 (21.8)	
Four to six times per week	38 (44.2)	48 (55.8)	86 (43.7)	
More than six times per week	31 (58.5)	22 (41.5)	53 (26.9)	
Reason to never eat meat	8 (53.3)	7 (46.7)	15 (100)	0.689
Environmental impact	4 (66.7)	2 (33.3)	6 (40.0)	
Being a strict vegetarian	3 (42.9)	4 (57.1)	7 (46.7)	
Other	1 (50.0)	1 (50.0)	2 (13.3)	
Willingness to reduce meat consumption due to health impact	85 (47.2)	95 (52.8)	180 (100)	0.898
Yes	66 (47.5)	73 (52.5)	139 (77.2)	
No	19 (46.3)	22 (53.7)	41 (22.8)	
Knowledge of the ecological footprint of meat	92 (48.2)	99 (51.8)	191 (100)	0.303
Yes	27 (42.9)	36 (57.1)	63 (33.0)	
No	65 (50.8)	63 (49.2)	128 (67.0)	
Willingness to reduce meat consumption due to environmental impact	88 (47.8)	96 (52.2)	184 (100)	0.379
Yes	57 (45.6)	68 (54.4)	125 (67.9)	
No	31 (52.5)	28 (47.5)	59 (32.1)	

The results are expressed as the number of individuals (%). Statistically significant (*p* < 0.05).

**Table 4:** Characterization of meat consumption and willingness to reduce its consumption by sex.

Characteristics	Male (*n* = 66)	Female (*n* = 131)	Total (*n* = 197)	*p*
Frequency of meat consumption	66 (35.5)	131 (66.5)	197 (100)	0.072
Never	1 (6.7)	14 (93.3)	15 (7.6)	
One to three times per week	12 (27.9)	31 (72.1)	43 (21.8)	
Four to six times per week	34 (39.5)	52 (60.5)	86 (43.7)	
More than six times per week	19 (35.8)	34 (64.2)	53 (26.9)	
Reason to never eat meat	1 (6.7)	14 (93.3)	15 (100)	0.448
Environmental impact	1 (16.7)	5 (83.3)	6 (40.0)	
Being a strict vegetarian	0 (0.0)	7 (100)	7 (46.7)	
Other	0 (0.0)	2 (100)	2 (13.3)	
Willingness to reduce meat consumption due to health impact	61 (33.9)	119 (66.1)	180 (100)	<0.001 *
Yes	37 ^a^ (26.6)	102 ^b^ (73.9)	139 (77.2)	
No	24 ^a^ (39.3)	17 ^b^ (14.3)	41 (22.8)	
Knowledge of the ecological footprint of meat	65 (34.0)	126 (66.0)	191 (100)	0.428
Yes	19 (30.2)	44 (69.8)	55 (38.4)	
No	46 (42.2)	82 (57.8)	109 (67.0)	
Willingness to reduce meat consumption due to environmental impact	63 (34.2)	121 (65.8)	184 (100)	<0.001 *
Yes	32 ^a^ (25.6)	93 ^b^ (74.4)	125 (67.9)	
No	31 ^a^ (52.5)	28 ^b^ (47.5)	59 (32.1)	

The results are expressed as the number of individuals (percentage). * Statistically significant (*p* < 0.05). Frequencies in the same row marked with different letters (a,b) are statistically different according to the Z test for proportions (*p* < 0.05).

**Table 5:** Association of availability to decrease the frequency of meat consumption in participants with consumption greater than six times per week.

Characteristics	Meat Consumption More than Six Times per Week					
	Yes (*n* = 53)	No (*n* = 144)	Total (*n* = 197)	*p*	OR	CI (95%)
Availability to reduce the frequency of meat consumption due to health impact	49 (27.2)	131 (72.8)	180 (100)	<0.001 *	3.612	1.73–7.55
Yes	29 ^a^ (20.9)	110 ^b^ (79.1)	139 (77.2)			
No	20 ^a^ (48.8)	21 ^b^ (51.2)	41 (22.8)			
Availability to reduce the frequency of meat consumption due to environmental impact	51 (27.7)	133 (72.3)	184 (100)	<0.001 *	4.006	2.02–7.93
Yes	23 ^a^ (18.4)	102 ^b^ (81.6)	125 (67.9)			
No	28 ^a^ (47.5)	31 ^b^ (52.5)	59 (32.1)			

The results are expressed as the number of individuals (percentage). CI, confidence interval; OR, odds ratio. * Statistically significant (*p* < 0.05). Frequencies in the same line marked with different letters (a,b) are statistically different according to the Z test for proportions (*p* < 0.05).

**Table 6:** Association by sex of availability to reduce the frequency of meat consumption in participants with a frequency of meat consumption greater than six times per week.

Characteristics	Meat Consumption More than Six Times per Week											
	M (*n* = 19)	*p*	OR	CI (95%)	F (*n* = 34)	*p*	OR	CI (95%)	Total (*n* = 53)	*p*	OR	CI (95%)
Availability to reduce the frequency of meat consumption due to health impact	19 (38.8)	0.388	1.620	0.534–4.865	30 (61.2)	<0.001 *	8.009	2.632–24.36	49 (100)	<0.001 *	3.612	1.73–7.55
Yes	10 (34.5)				19 (65.5)				29 (77.2)			
No	09 (45.0)				11 (55.0)				20 (22.8)			
Availability to reduce the frequency of meat consumption due to environmental impact	19 (37.3)	0.146	2.256	0.746–6.822	32 (62.7)	<0.001 *	6.417	2.55–16.13	51 (100)	<0.001 *	4.006	2.02–7.93
Yes	07 (30.4)				16 (69.6)				23 (45.1)			
No	12 (42.9)				16 (57.1)				28 (54.9)			

The results are expressed as the number of individuals (percentage). F, female; CI, confidence interval; M, male; OR, odds ratio. * Statistically significant (*p* < 0.05).

## Data Availability

Not applicable.
